# Dietary intake of fructose increases purine *de novo* synthesis: A crucial mechanism for hyperuricemia

**DOI:** 10.3389/fnut.2022.1045805

**Published:** 2022-12-19

**Authors:** Pengfei Zhang, Huimin Sun, Xinyu Cheng, Yajing Li, Yanli Zhao, Wuxuan Mei, Xing Wei, Hairong Zhou, Yunbo Du, Changchun Zeng

**Affiliations:** ^1^Department of Critical Care Medicine, Shenzhen Longhua District Central Hospital, Shenzhen, China; ^2^Department of Medical Laboratory, Shenzhen Longhua District Central Hospital, Shenzhen, China; ^3^Clinical Medical College, Hubei University of Science and Technology, Xianning, China; ^4^Department of Nephrotic Rheumatism, Shenzhen Longhua District Central Hospital, Guangdong Medical University, Shenzhen, China; ^5^Department of General Practice, Shenzhen Longhua District Central Hospital, Guangdong Medical University, Shenzhen, China

**Keywords:** purine *de novo* synthesis, fructose, hyperuricemia, metabolomic analysis, RNA-seq analysis

## Abstract

**Background:**

Fructose consumption is a potential risk factor for hyperuricemia because uric acid (UA) is a byproduct of fructose metabolism caused by the rapid consumption of adenosine triphosphate and accumulation of adenosine monophosphate (AMP) and other purine nucleotides. Additionally, a clinical experiment with four gout patients demonstrated that intravenous infusion of fructose increased the purine *de novo* synthesis rate, which implied fructose-induced hyperuricemia might be related to purine nucleotide synthesis. Moreover, the mechanistic (mammalian) target of rapamycin (mTOR) is a key protein both involved in fructose metabolism and purine *de novo* synthesis. The present study was conducted to elucidate how fructose influences mTOR and purine *de novo* synthesis in a hepatic cell line and livers of mice.

**Materials and methods:**

RNA-sequencing in NCTC 1469 cells treated with 0- and 25-mM fructose for 24 h and metabolomics analysis on the livers of mice fed with 0- and 30-g/kg fructose for 2 weeks were assessed. Gene and protein expression of phosphoribosyl pyrophosphate synthase (PRPSAP1), Glutamine PRPP aminotransferase (PPAT), adenyl succinate lyase (ADSL), adenyl succinate synthetase isozyme-1 (Adss1), inosine-5’-monophosphate dehydrogenase (IMPDH), and guanine monophosphate synthetase (GMPS) was measured. The location of PRPSAP1 and PPAT in the liver was assessed by an immunofluorescence assay.

**Results:**

Metabolite profiling showed that the level of AMP, adenine, adenosine, hypoxanthine, and guanine was increased significantly. RNA-sequencing showed that gene expression of phosphoribosyl pyrophosphate synthase (PRPS2), phosphoribosyl glycinamide formyl transferase (GART), AICAR transformylase (ATIC), ADSL, Adss1, and IMPDH were raised, and gene expression of adenosine monophosphate deaminase 3 (AMPD3), adenosine deaminase (ADA), 5’,3’-nucleotidase, cytosolic (NT5C), and xanthine oxidoreductase (XOR) was also increased significantly. Fructose increased the gene expression, protein expression, and fluorescence intensity of PRPSAP1 and PPAT in mice livers by increasing mTOR expression. Fructose increased the expression and activity of XOR, decreased the expression of uricase, and increased the serum level of UA.

**Conclusion:**

This study demonstrated that the increased purine *de novo* synthesis may be a crucial mechanism for fructose-induced hyperuricemia.

## Introduction

Hyperuricemia is an excessively increased uric acid (UA) level in blood. The upper limit of normal is 6.8 mg/dL (> 7 mg/dL can lead to symptoms). A high blood level of UA is associated with gout, renal dysfunction, diabetes mellitus, hypertension, and atherosclerosis (1–3). In general, hyperuricemia is caused by urate overproduction, typically due to consumption of a purine-rich diet (e.g., beer, meat, seafood), high cell turnover, or urate excretion disorders (4). However, fructose and alcohol can also increase the serum UA level, and have gained more and more attention as a potential risk factor for hyperuricemia (especially fructose) (5).

Fructose is a primary sweetener used in various industrial food products, and is also present in fresh fruits and corn syrup (6). Fructose intake has increased markedly over recent years, and consumption has exceeded physiological needs (7). In liver, fructose can be metabolized more readily than glucose because of a specific enzyme (fructokinase), which catalyzes the conversion of fructose to fructose-1-phosphate using adenosine triphosphate (ATP) as a phosphate donor (8). Fructokinase is not regulated and phosphorylates fructose as rapidly as it can, leading to depletion of intracellular ATP to generate adenosine monophosphate (AMP). AMP accumulation stimulates AMP deaminase, which results in degradation of purine nucleotide (PNs) to UA, and increases the serum UA level (9, 10): this is a well-known mechanism of fructose-induced hyperuricemia. If so, more rapid depletion of ATP induced by fructose intake only provides the precursors (PNs) for UA synthesis, which would be terminated due to fructose depletion. However, consumption of 8% fructose drinking for 8 weeks has been shown to induce stable and persistent hyperuricemia in animal models, suggesting a long-time chronic effect of fructose upon hyperuricemia (4).

Fructose intake also stimulates UA biosynthesis from amino acid precursors (1, 11). Furthermore, a clinical experiment with four gout patients demonstrated that intravenous infusion of fructose increased the rate of purine *de novo* synthesis, which implied that the mechanism of fructose-induced hyperuricemia might be related to purine nucleotide synthesis (12).

Mammalian target of rapamycin (mTOR) is a key protein involved in the metabolic effect of fructose (13). Moreover, mTOR has been reported to promote expression of the genes related to the biosynthesis of phosphoribosyl pyrophosphate (the substrate for the first reaction in the purine *de novo* synthesis) (14). Thus, understanding the regulatory effect of mTOR on purine *de novo* synthesis may provide new ideas for the rational intake of fructose as well as the prevention and treatment of metabolic diseases induced by excessive intake of fructose.

We postulated that the mechanism of fructose-induced hyperuricemia was related to the purine nucleotide synthesis and purine degradation in the liver. The present study investigated the effect of giving fructose (20, 30, and 40 g/kg, p.o.) for 2 weeks to induce a high serum-level of UA. Then, RNA-sequencing analysis and metabolomics analysis were used to ascertain the effect of fructose upon the *de novo* synthesis of purine on hepatocytes and the liver of mice. The results showed that fructose intake increased the activity of phosphoribosyl pyrophosphate synthase (PRPSAP1) and glutamine PRPP aminotransferase (PPAT) to accelerate the *de novo* synthesis of purine to inosine 5’-monophosphate (IMP), and drove IMP to synthesize AMP, thereby accelerating its catabolism to UA by increasing the activity of xanthine oxidoreductase (XOR). This study demonstrated increased purine *de novo* synthesis as a crucial mechanism for fructose-induced hyperuricemia.

## Materials and methods

### Cell culture and fructose treatment

NCTC 1469 cells (catalog number: CL-0407; Procell Life Science and Technology, Wuhan, China) were cultured in glucose (4.5 g/L)-containing Dulbecco’s modified Eagle’s medium with 10% (v/v) fetal bovine serum, and 1% penicillin/streptomycin solution (100 units/mL penicillin and 100 μg/mL streptomycin) in an atmosphere of 5% CO_2_ and 95% humidified air at 37°C.

For fructose (purity = 99%; F0127; MilliporeSigma, Burlington, MA, USA) treatment, cells were seeded on six-well plates (6 × 10^5^ cells per well). After reaching 80% confluence, cells were treated with medium containing fructose (0 and 25 mM) for 24 h. Then, cells were harvested for messenger (m)RNA extraction and RNA-sequencing. For the protein expression determination by western blotting, cells were seeded on six-well plates (6 × 10^5^ cells per well) and treated with medium containing fructose (0, 1.25, 2.5, 5.0, 10, and 20 mM) for 24 h. For the mechanism detection, cells were treated with medium containing fructose (10 and 20 mM) in the presence or absence of Rapamycin at 200 nM.

### RNA-sequencing

Cells in six-well plates were harvested after 24 h of treatment for extraction of total RNA using TRIzol^®^ Reagent (254712; Ambion Life Technologies, Carlsbad, CA, USA). mRNA was enriched with poly-A selection, and 50 base pair paired-end RNA-sequencing was completed on the BGISEQ platform at Shenzhen SunV Biotech (Shenzhen, China). Raw reads were filtered using SOAP and SOAPnuke (15), Clean reads were mapped to the transcriptome of the RefSeq database using Bowtie2 (16). Gene expression was counted by RSEM (17) and normalized as transcripts per kilobase of exon model per million mapped reads. DESeq2 was employed to evaluate differential expression. Differentially expressed genes (DEGs) were identified by Benjamini Hochberg-adjusted *P* value (< 0.05) (18). Analyses of enrichment of function and metabolic pathways were undertaken based on Gene Ontology (GO^1^) and Kyoto Encyclopedia of Genes and Genomes Kyoto Encyclopedia of Genes and Genomes (KEGG^2^) databases using Database for Annotation, Visualization and Integrated Discovery^3^ DAVID (19).

### Animals and treatments

This study protocol was approved by the Animal Care and Use Committee of Guangdong Medical University (Zhanjiang. China).

Male C57BL/6 wild-type mice (6 weeks, 20–25 g, specific pathogen-free) were obtained from the Experimental Animal Center of Guangdong Medical University [production license: SCXK (Yue) 2018-0008] and allowed to acclimatize to the animal facility environment for 1 week before experimentation. Mice were maintained in a specific pathogen-free, temperature (24 ± 2°C)-and humidity (50 ± 5%)-controlled environment, with a standard 12 h light/dark cycle. A total of twenty-four mice were divided randomly into 4 treatment groups, including one control (mice were given distilled water) and three fructose groups (20, 30, 40 g/kg bodyweight). Each of these groups consisted of 6 mice. Fructose was prepared as a solution at 1, 1.5, and 2 g/mL concentrations. The solution was given *via* oral route twice daily at the volume of 0.5 mL (0.25 mL each time at 9:00 a.m. and 3:00 p.m.). Mice feed and water were provided *ad libitum* throughout the experiment. Additionally, the samples were collected 48 h after experiment cessation.

### Uric acid level in blood

Approximately 1 mL of blood samples were collected from the angular vein of mice under anesthesia. Serum samples were obtained by centrifugation at 1,790 × *g* for 10 min at room temperature and stored in microtubes at −80°C. The UA level was determined using a commercially available kit purchased from Solarbio Life Science (Beijing, China), following the manufacturer’s instructions.

### Liver collection and metabolomics analysis

Each mouse’s liver was harvested and washed with ice-cold, sterile 0.9% saline solution to remove blood contamination. Then, it was placed in a cryogenic vial. Liquid chromatography-mass spectrometry (LC-MS) was conducted at BioNovoGene (Suzhou, China). In brief, ∼20 mg of liver from both the control group and the 30 g/kg fructose group, respectively, was collected and placed in a centrifuge tube containing magnetic beads and ground to a homogenate using 1 mL of homogenate medium (80% methanol). Then, the homogenate was placed in liquid nitrogen for 30 s and ice for 5 min (three times). The extract was obtained by centrifugation at 12,000 × *g* for 10 min at 4°C and subjected to LC-MS with an ACQUITY UPLC^®^ HSS T3 column (1.8 μm, 2.1 × 150 mm, Ethylene Bridged Hybrid, Waters, Milford, MA, USA) (20). Samples were randomized, data acquisitions were done in one batch to eliminate system errors, and the metabolites were identified based on their molecular weight, mass spectra, and retention time. Original LC-MS data were processed and analyzed using Bio-deep Online software^4^ with optimized settings. Annotation of metabolite using LC-MS data was done with the Compound Discover 3.3 (Thermo Scientific, Waltham, MA, USA) and referenced to the mzCloud database.^5^ Discrimination metabolites between two classes of samples was done using a statistically significant threshold of Variable Importance in Projection (VIP) value (VIP > 1) and validated further by Student’s *t*-test analysis (*P* ≤ 0.05). Principal components analysis (PCA) and partial least squares discriminant analysis (PLS-DA) were carried out with SIMCA-P software. Heatmaps were constructed using Euclidian distances and complete linkage grouping with the “heatmap” package in R language (R Institute for Statistical Computing, Vienna, Austria). Relative quantitative values of metabolites were normalized, transformed, and clustered through hierarchical clustering. Metabolite correction was assessed using Pearson’s correction coefficient and constructed Cytoscape 3.2.7.^6^ To further identify alternative metabolic pathways, differential metabolites were subjected to grouping and enrichment of metabolic pathways using MetaboAnalyst 4.0^7^ and the KEGG database. Univariate analysis of Variance (ANOVA) was used to determine the significance of differences in contents between different groups.

### Quantitative real-time PCR

mRNA expression of PRPSAP1, PPAT, and XOR was determined with the SYBR Green II Real-Time PCR kit (Thermo Fisher Scientific). Total RNA was extracted from NCTC 1469 cells and mouse livers using the RNAiso Plus kit (TaKaRa Biotechnology, Dalian, China) following manufacturer instruction. RNA integrity was checked *via* agarose gel electrophoresis with ethidium bromide. The concentration and purity of RNA were determined using an automatic microplate reader (Synergy H4, BioTek, Tokyo, Japan) at an OD 260/280 reading ratio of 1.8–2.1. After determination of the RNA concentration, total RNA (1 μg) was reverse-transcribed into complementary DNA (cDNA) using the Prime Script RT Reagent Kit (TaKaRa Biotechnology). Real-time PCR was carried out with an ABI StepOnePlus real-time PCR system (Applied Biosystems, Grand Island, Foster City, CA, USA) using the SYBR Premix Ex Taq II Reagent Kit (TaKaRa Biotechnology) under manufacturer instructions but with modification (21). The volume of the reaction system was 20 μL [10 μL of SYBR Premix Ex Taq∐, 0.4 μL of PCR forward primer (10 μM), 0.4 μL of PCR reverse primer (10 μM), 2 μL of cDNA, and 7.2 μL of RNase-free H_2_O]. The primer sequences of target genes and reference genes were obtained from GenBank and are shown in Table 1. Relative level of mRNA expression was calculated using the 2^–ΔΔCT^ method after normalization against the reference gene (glyceraldehyde 3-phosphate dehydrogenase; GAPDH) (22).

**TABLE 1:** Primer sequences used for real-time RT-qPCR.

Primer	Forward 5′—3′	Reverse 5′—3′
PRPSAP1	ACTTATCCCAGAAAATCGCTGAC	CCACACCCACTTTGAACAATGTA
PPAT	GCGAGGAATGTGGTGTGTTTG	TTTAGGCACTGCACTCCCATC
XOR	CCGCCTTCAGAACAAGATCG	CCTTCCACAGTTGTCACAGC
HPRT	CTTCCTCCTCAGACCGCTTTT	AGCAAGTCTTTCAGTCCTGTCC
APRT	CCCGGGATTGACGTGAGTTT	GAGGGGCGAGATATCCCTGA
UOX	CAGATGAGAAACGGACCTCCC	GCCGTAGGGATTGTCGAGAG
GAPDH	CATCACTGCCACCCAGAAGACTG	ATGCCAGTGAGCTTCCCGTTCAG

### Western blotting

Primary antibodies against PRPSAP1 (catalog number: bs-19409R) and PPAT (bs-6359R) were purchased from Beijing Biosynthesis Biotechnology (Beijing, China). Antibody against phosphorylated (P) P-mTOR (Ser 2448, 5536S) was obtained from Cell Signaling Technology (Danvers, MA, USA). Antibodies against adenyl succinate synthetase isozyme 1 (AdSS1; sc-166401), adenyl succinate lyase (ADSL; sc-365623), guanine monophosphate synthetase (GMPS; sc-376163), and inosine-5’-monophosphate dehydrogenase (IMPDH; sc-166551) were sourced from Santa Cruz Biotechnology (Houston, TX, USA).

Liver samples were frozen in liquid nitrogen, ground into powder in a mortar, and lysed by addition of RIPA lysis buffer (Beyotime Biotechnology, Jiangsu, China). The NCTC 1469 cell lysates were prepared by harvesting cells in RIPA lysis buffer. The supernatant was centrifuged at 12,000 × *g* for 15 min at 4°C after samples had been lysed fully. Supernatants containing total proteins were quantified with a Bicinchoninic Acid Protein Kit (Meilun Biotechnology, Dalian, China); 50 μg of whole protein samples were resolved with 8, 10, or 15% polyacrylamide gel (depending on the molecular size of the proteins to be analyzed). Thereafter, immunoblotting was done by transferring resolved proteins onto polyvinylidene difluoride (PVDF) membranes in trans-buffer at 100 V for 1 or 2 h, depending on the molecular size of the protein. PVDF membranes were blocked with 5% skimmed milk or 5% bovine serum albumin (for phosphorylated protein) in TBST buffer [Tris–HCl (20 mM), pH 7.5; sodium chloride (150 mM); 0.05% Tween 20] for 2 h, washed thrice with TBST buffer for 5-min each time, and incubated overnight with primary antibodies (1:1000 dilution) at 4°C. Afterward, PVDF membranes were washed thrice with TBST buffer for 5-min each time and incubated with anti-rabbit (or anti-mouse) secondary antibodies conjugated to horseradish peroxidase (1:2000 dilution) for 2 h at room temperature. Blots were developed in the dark using an electrochemiluminescence detection kit. Developed blots were subjected to densitometric analysis using ImageJ 1.45 (US National Institutes of Health, Bethesda, MD, USA) and expression normalized to that of GAPDH (D16H11; Cell Signaling Technology) as the internal control.

### Immunohistochemistry

Paraffin-embedded liver tissues were used to measure expression of PRPSAP1 and PPAT. After deparaffinization, slides were heated in an autoclave with sodium citrate (for antigen repair), followed by 1% hydrogen peroxide (to abolish endogenous peroxidase activity), and blocked with 2% goat serum. Then, slides were incubated with primary antibodies PRPSAP1 and PPAT at 1:200 dilution overnight at 4°C, followed by incubation with fluorescein isothiocyanate-conjugated goat anti-rabbit immunoglobin G (1:500 dilution; Beyotime Biotechnology) for 2 h at room temperature. Slides were counterstained with 4’,6-diamidino-2-phenylindole for 5 min to stain nuclei. Coverslips were mounted on slides followed by visualization under a confocal laser scanning microscope (LSM 800, Carl Zeiss, Wetzlar Germany) using laser set at 405 and 488 nm.

### Hepatic xanthine oxidoreductase activity

Approximately 0.5 g of liver tissue was used to prepare homogenates. Minced tissue was homogenized in an ice-cold phosphate buffer solution (*w/v*, 1:9) using a glass homogenate tube. The tube centrifuged at 8,000 × *g* for 10 min at 4°C. The supernatant was collected and stored at −80°C until further analyses. XOR activity was estimated using a kit (Solarbio Life Science), as reported previously (23). The protein concentration in tissue homogenates was determined using the Bicinchoninic Acid Protein Kit (24).

### Statistical analysis

Quantitative data are presented as the mean ± SD of three independent experiments. Results were evaluated *via* one-way ANOVA followed by Tukey’s multiple-comparison test using Prism 6 (GraphPad, San Diego, CA, USA). *P* < 0.05 was considered significant.

## Results

### Fructose consumption increases hepatic purine synthesis

**FIGURE 1:**
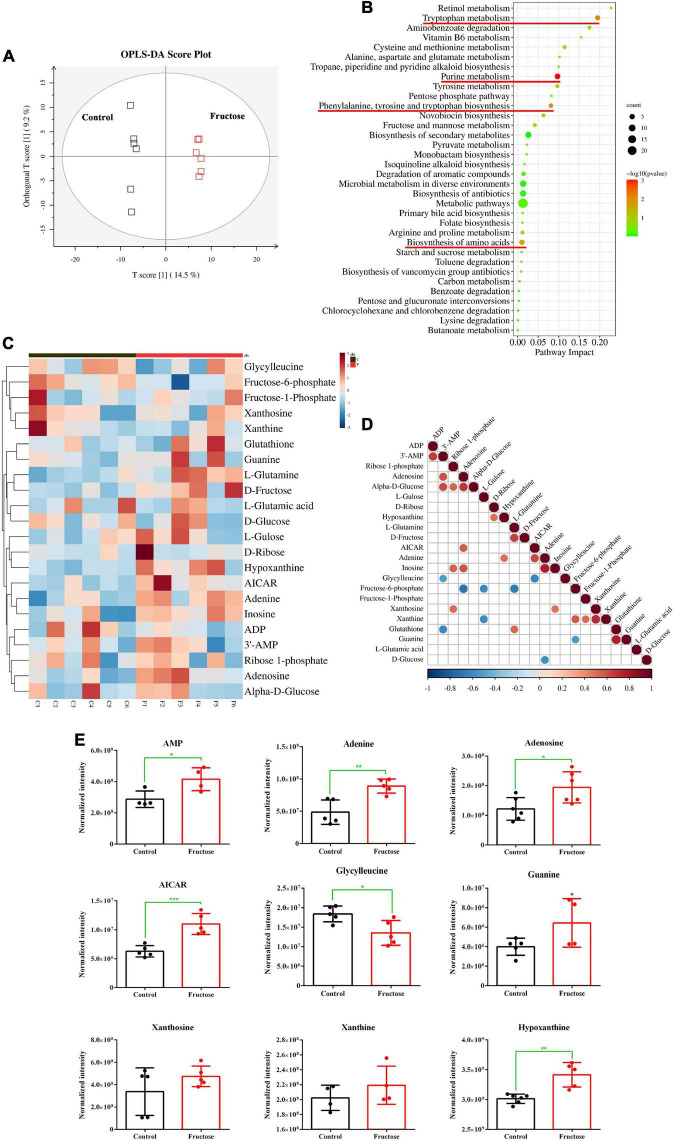
Fructose consumption increases hepatic purine synthesis **(A)** partial least squares discriminant analysis (PLS-DA) score plots for the samples remarkably separated the fructose group from the control groups. **(B)** Pathway enrichment analysis showed the pathways most significantly altered in the fructose compared with the control group (the significantly altered metabolic pathways were marked with the red line, *P* < 0.05). **(C)** Heat map of the differentially expressed metabolites related to *de novo* purine synthesis between fructose group and control group. **(D)** Thermogram of metabolite association analysis, the orange-red points represent a positive correlation between two metabolites (*P* < 0.05), and the blue-green points represent a negative correlation between two metabolites (*P* < 0.05). **(E)** The normalized intensity of 9 metabolites of purine synthesis and decomposition. The metabolite profiling was performed in mouse liver. Data are expressed as mean ± SD (*n* = 6). *P < 0.05, **P < 0.01, ***P < 0.001 (*represents 30 g/kg fructose groups compared with the control group).

Twelve samples (six samples from the control group and six samples from the 30-g/kg fructose group) were analyzed. Untargeted metabolomics analysis was done by LC-MS; 12 valid samples were identified and measured. Plots for PLS-DA score between the control group and fructose group were clearly different (Figure 1A), which suggested that metabolic biomarkers could be selected. Analysis of enrichment of metabolic pathway was conducted: enrichment was observed in “purine metabolism,” “tryptophan metabolism,” “phenylalanine, tyrosine, and tryptophan biosynthesis,” and “biosynthesis of amino acids” (Figure 1B). Twenty-two metabolites related to purine synthesis were identified as having differential expression in the fructose group compared with the control group (Figure 1C). Fifteen metabolites were upregulated (including AMP, adenine, and adenosine) and seven metabolites were downregulated (including fructose-6-phosphate, glycylglycine, and L-glutamic acid). Correlation analysis of these metabolites showed no correlation between fructose-1-phosphate and the PNs (AMP, adenine, and adenosine) (Figure 1D). However, a negative correlation between glycylglycine and AMP, and a positive correlation between 5-Aminoimidazole-4-carboxamide1-β-D-ribofuranoside (AICAR) and adenosine, were found (Figure 1D). We explored potential diagnostic biomarkers for purine synthesis. The significantly increased metabolites of AMP, adenine, adenosine, AICAR, hypoxanthine, and guanine indicated upregulation of purine *de novo* synthesis (Figure 1E). Based on metabolites profiling and model evaluation, fructose intake appeared to upregulate the purine synthesis.

### Differentially expressed genes and differentially expressed pathways between fructose and the control group determined by transcriptomic analysis

**FIGURE 2:**
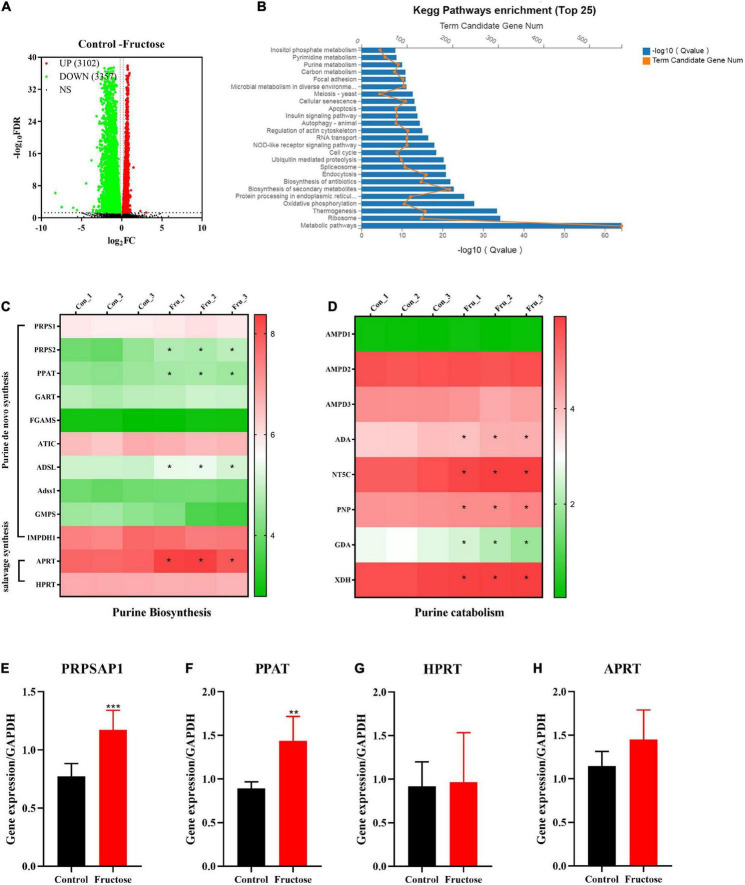
Differentially expressed genes (DEGs) and differentially expressed pathways between fructose and the control group determined by transcriptomic analysis. **(A)** Volcano plot showed the DEGs were screened between the fructose group and the control group in accordance with the corresponding BGISEQ platform. The threshold was set to log_2_| FC| > 1 and *P* < 0.05. **(B)** Pathway enrichment analysis showed the top 25 metabolism pathways most significantly altered in the fructose compared with the control group. **(C,D)** Heat map revealed gene expression differences related to purine biosynthesis and catabolism (*P < 0.05 represents the fructose group compared with the control group). **(E–G)** And **(H)** qPCR validation results of phosphoribosyl pyrophosphate synthase (PRPSAP1), Glutamine PRPP aminotransferase (PPAT), hypoxanthine-guanine phosphoribosyl transferase (HPRT), and adenine phosphoribosyl transferase (APRT). The RNA sequential analysis was performed in the NCTC1469 cells. Data are expressed as mean ± SD (*n* = 3). *P < 0.05, **P < 0.01, ***P < 0.001 (*represents 25 mM fructose groups compared with the control group).

Six samples (three samples in each group) of NCTC1469 cells were used for transcriptomic analysis. Transcriptomic data were downloaded from BGI with the corresponding BGISEQ platform. A total of 6,459 genes were selected as DEGs between fructose group and control group (Figure 2A). The top-25 enriched pathways according to the KEGG database included “purine metabolism,” “carbon metabolism,” and “pyrimidine metabolism” (Figure 2B): these pathways are associated with purine *de novo* synthesis. To further identify the role of fructose on purine synthesis, thirteen genes (purine synthesis) and eight genes (purine catabolism) were chosen for analysis. Expression of the genes involved in purine *de novo* synthesis [e.g., PRPS, phosphoribosyl glycinamide formyl transferase (GART), AICAR transfomylase (ATIC), ADSL, Adss1, and IMPDH (Figure 2C)] and purine catabolism [e.g., adenosine monophosphate deaminase 3 (AMPD3), adenosine deaminase (ADA), 5’, 3’-nucleotidase, cytosolic (NT5C), and XOR] was upregulated significantly (Figure 2D). RT-qPCR showed that fructose (25 mM) consumption increased the gene expression of PRPSAP1 (Figure 2E) and PPAT (Figure 2F) significantly, but had no impact on the gene expression of hypoxanthine-guanine phosphoribosyl transferase (HPRT) (Figure 2G) or adenine phosphoribosyl transferase (APRT) (Figure 2H). These results suggested that fructose accelerated the *de novo* synthesis of purine, while promoting the catabolism of purine into UA.

### Fructose increases hepatic expression of phosphoribosyl pyrophosphate synthase and Glutamine PP aminotransferase

**FIGURE 3:**
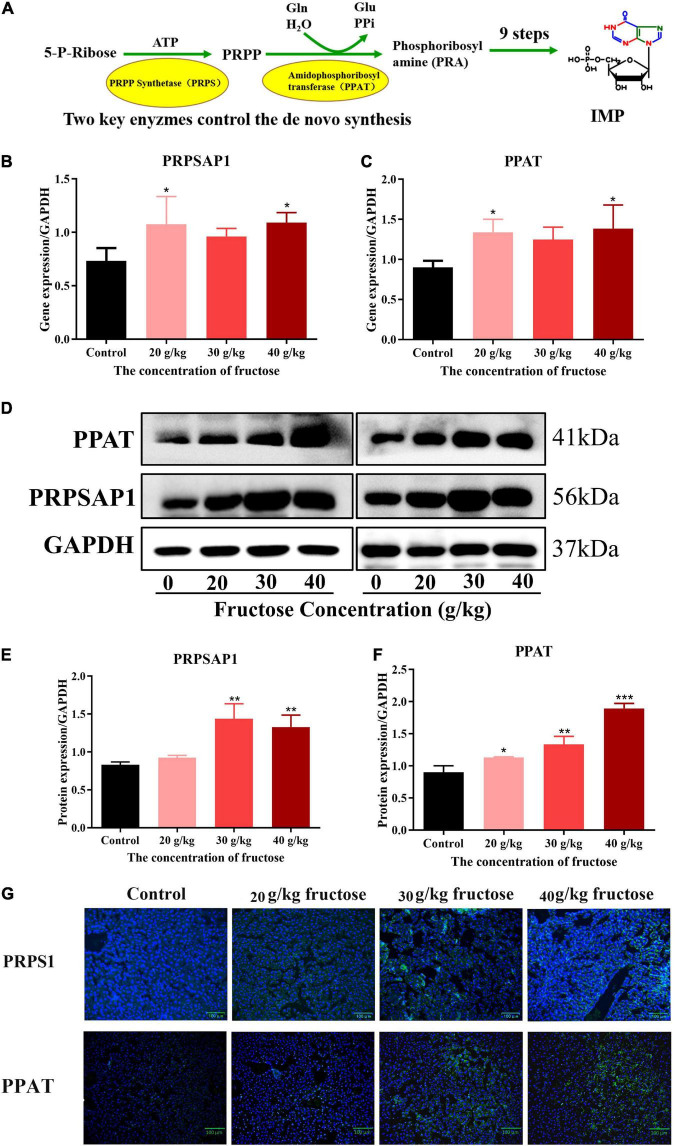
Fructose increases hepatic expression of phosphoribosyl pyrophosphate synthase (PRPSAP1) and Glutamine PRPP aminotransferase (PPAT). **(A)** The key enzymes control the *de novo* synthesis. **(B,C)** Gene expression of PRPSAP1, PPAT in reference to glyceraldehyde 3-phosphate dehydrogenase (GAPDH). **(D)** Immunoblot analysis for PRPSAP1 and PPAT in reference to GAPDH. **(E,F)** The relative protein expression of PRPSAP1 and PPAT in regard to GAPDH. **(G)** Immunofluorescence analysis for PRPSAP1 and PPAT in the liver. Scale bars: 100 μm. Data are expressed as mean ± SD (*n* = 6). *P < 0.05, **P < 0.01, ***P < 0.001 (*represents the fructose group compared with the control group).

Liquid chromatography-mass spectrometry (LC-MS) and RNA-sequencing data suggested that fructose could accelerate the purine *de novo* synthesis. To confirm this finding, expression of PRPSAP1 and PPAT (Figure 3A) was determined in the livers of fructose-fed mice. Gene expression of PRPSAP1 and PPAT was upregulated significantly by treatment with fructose (20, 40 g/kg) (*P* < 0.05, Figures 3B,C). With respect to hepatic protein expression, treatment with fructose (30, 40 g/kg) stimulated PRPSAP1 expression significantly (*P* < 0.05, Figures 3D,E), and PPAT expression in all three fructose groups was higher than that in the control group (*P* < 0.05) (Figures 3D,F). With an increase in the fructose concentration, the fluorescence intensity of PRPSAP1 and PPAT in liver slices increased gradually (Figure 3G). Taken together, these results demonstrated the promoting effect of fructose on the purine *de novo* synthesis.

### Fructose accelerates purine *de novo* synthesis by increasing Mammalian target of rapamycin expression

**FIGURE 4:**
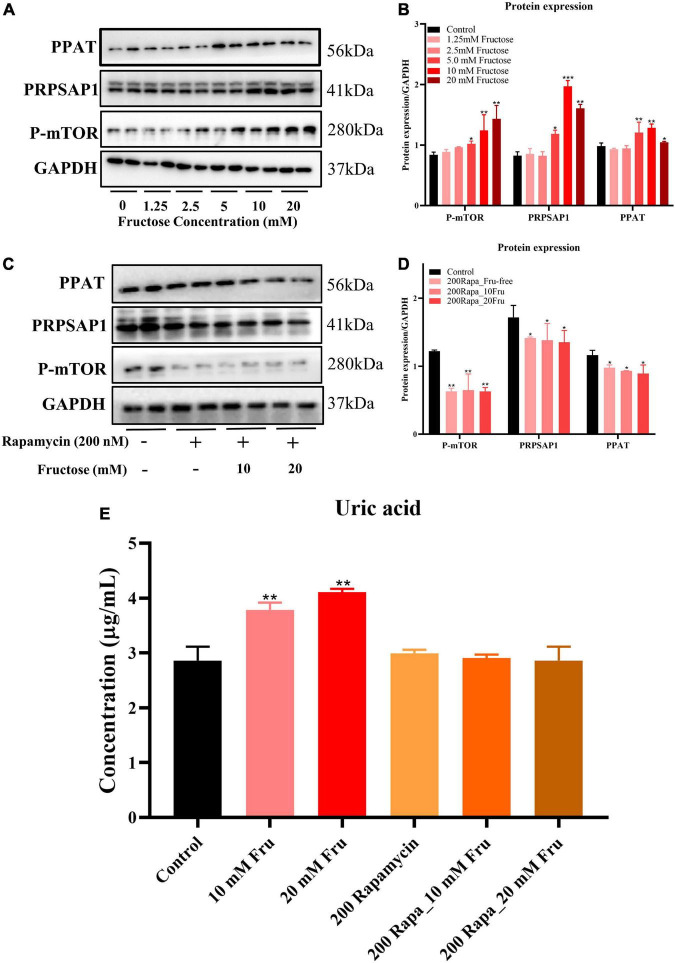
Fructose accelerates purine *de novo* synthesis by increasing mammalian target of rapamycin (mTOR) expression. **(A,B)** Immunoblot analysis for P-mTOR, phosphoribosyl pyrophosphate synthase (PRPSAP1), and Glutamine PRPP aminotransferase (PPAT) in fructose-treated NCTC1469 cells. **(C,D)** Immunoblot analysis for P-mTOR, PRPSAP1, and PPAT in the presence of rapamycin. **(E)** The uric acid changes in the supernatant of fructose-treated NCTC 1469 cells. Data are expressed as mean ± SD (*n* = 6). *P < 0.05, **P < 0.01, ***P < 0.001 (*represents the fructose group compared with the control group).

An *in vitro* experiment was conducted to investigate how fructose treatment increases expression of PRPSAP1 and PPAT. Fructose treatment increased protein expression of P-mTOR, PRPSAP1, and PPAT in a dose-dependent manner, and significant effects were observed at concentrations of 5, 10, and 20 mM (*P* < 0.05) (Figures 4A,B). The presence of rapamycin inhibited P-mTOR expression significantly, and protein expression of PRPSAP1 and PPAT was reduced compared with that in the control group (*P* < 0.05) (Figures 4C,D). Fructose treatment did not affect protein expression of PRPSAP1 or PPAT in the presence of rapamycin (*P* > 0.05) (Figures 4C,D). Fructose treatment increased the UA level in cell supernatants significantly at concentrations of 10 and 20 mM (*P* < 0.05) (Figure 4E), but the UA level was not affected by fructose in the presence of rapamycin (*P* > 0.05) (Figure 4E). Taken together, these results suggested that fructose accelerated purine *de novo* synthesis by increasing P-mTOR expression.

### Fructose promoted inosine 5’-monophosphate to adenosine monophosphate conversion and the catabolism of adenosine monophosphate to uric acid

**FIGURE 5:**
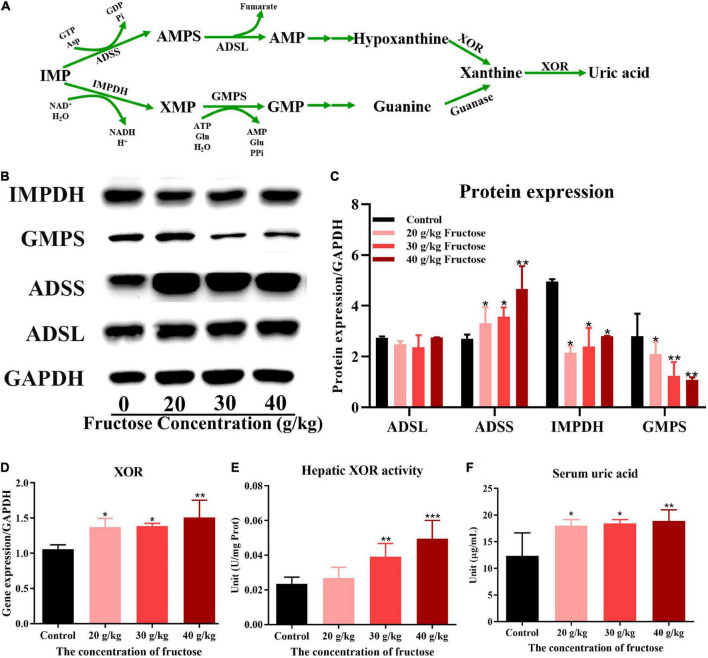
Fructose promoted inosine 5’-monophosphate (IMP) to adenosine monophosphate (AMP) conversion and the catabolism of AMP to uric acid (UA). **(A)** The processes of synthesis and catabolism of AMP and guanine monophosphate (GMP). **(B)** Immunoblot analysis for adenyl succinate synthetase isozyme (ADSS), adenyl succinate lyase (ADSL), guanine monophosphate synthetase (GMPS), inosine-5’-monophosphate dehydrogenase 1 (IMPDH) in reference to GAPDH. **(C)** The relative protein expression of adenyl succinate lyase (ADSL), adenyl succinate synthetase (ADSS), inosine-5’-monophosphate dehydrogenase (IMPDH), and guanine monophosphate synthetase (GMPS) in regard to lyceraldehyde 3-phosphate dehydrogenase (GAPDH). **(D)** The gene expression of hepatic xanthine oxidoreductase (XOR) in reference to GAPDH. **(E)** The activity of xanthine oxidoreductase in the liver. **(F)** Changes in serum uric acid level in fructose-fed mice. Data are expressed as mean ± SD (*n* = 6). *P < 0.05, **P < 0.01, ***P < 0.001 (*represents the fructose group compared with the control group).

**FIGURE 6:**
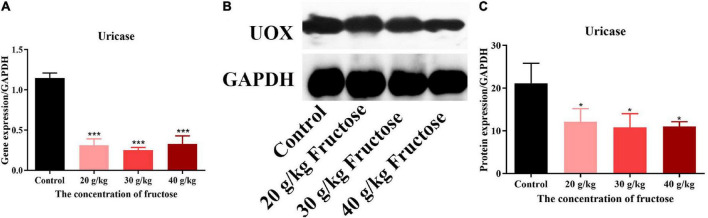
Fructose inhibited the gene and protein expression of uricase. **(A)** The gene expression of uricase (UOX) in liver. **(B)** Immunoblot analysis of UOX. **(C)** The relative protein expression of UOX in reference to GAPDH. Data are expressed as mean ± SD (*n* = 6). *P < 0.05, **P < 0.01, ***P < 0.001 (*represents the fructose group compared with the control group).

Fructose appeared to increase IMP synthesis by promoting *de novo* synthesis of purine, but the effect of fructose on the synthesis of IMP to AMP, or guanine nucleotide (GMP) (Figure 5A) is not known. All three concentrations of fructose enhanced the expression of ADSS (*P* < 0.05) (Figures 5B,C), but did not affect the expression of ADSL. However, fructose decreased expression of IMPDH and GMPS in the liver (*P* < 0.05) (Figures 5B,C). These findings suggested that fructose drives IMP to synthesize AMP, not GMP. The final product of AMP decomposition is UA, and XOR is the crucial enzyme controlling this process (Figure 5A). Fructose increased the gene expression (Figure 5D) and the activity (Figure 5E) of XOR in the liver as compared with that in the control group (*P* < 0.05). Otherwise, fructose intake decreased the gene expression (Figure 6A) and protein expression (Figures 6B,C) of uricase significantly. Taken together, these data suggested that fructose consumption increased the serum UA level (*P* < 0.05) (Figure 5F) and that fructose drives IMP to synthesize AMP and accelerates its catabolism to UA by increasing XOR activity.

## Discussion

Conventional thinking dictates that rapid metabolism of fructose can induce ATP consumption to generate AMP, and that AMP accumulation stimulates AMP deaminase, thereby resulting in PNs degradation to UA (9, 10). Furthermore, the possible mechanism related to purine metabolism is: (i) increased synthesis of (*de novo* and salvage) of PNs, (ii) increased breakdown of preformed PNs and PN derivatives, (iii) a combination of (i) and (ii).

Metabolite profiling in mice livers showed an increase in levels of PNs and their derivatives, (AMP, adenine, adenosine, guanine, xanthosine, xanthine, hypoxanthine) (Figure 1E) induced by fructose intake, which suggested that increased breakdown of PNs and their derivatives was operative after fructose administration. Fructose intake can result in stimulation of *de novo* synthesis of purine, so the change in ribose-5-phosphate (R5P) level must be determined first (25). However, the metabolic results failed to show an increase in R5P level in the mice livers during fructose administration. In addition, metabolic intermediates can also serve to control metabolism. AICAR is a critical signaling intermediate within purine *de novo* synthesis pathway, and its expression is increased significantly by fructose intake. Increased AICAR level can promote the ATIC activity, which accelerates the last final two steps in the pathway to convert AICAR to IMP (14).

The non-essential amino acid glycine contributes carbon and nitrogen atoms at positions of C4, C5, and N6 in purine ring at the beginning of purine synthesis (25). Our metabolic data showed a significant decrease in glycine level induced by fructose consumption, so glycine was used rapidly as a supplier of carbon atoms. Combination of metabolic data suggested that fructose administration accelerated purine synthesis as well as the breakdown of preformed PNs and their derivatives in the livers of fructose-fed mice.

Under normal physiological conditions, the cellular “purine pool” is derived primarily from recycling of degraded bases *via* the salvage pathway. Nevertheless, the pathway related to purine *de novo* synthesis will be upregulated to meet demand if cells require higher levels of purine (26). Purine *de novo* synthesis occurs in 10 steps, generating IMP from PRPP by sequential orchestration of six enzymes. Transcriptomics analysis in the present study showed that gene expression of PRPS2, PPAT, GART, and ATIC was upregulated upon fructose intake, which can accelerate the pathway for purine *de novo* synthesis. For salvage synthesis, gene expression of APRT and HPRT was not affected significantly by fructose intake. This finding suggested that fructose administration increased purine *de novo* synthesis instead of salvage synthesis in the livers of mice. The significantly increased gene expression of AMPD3, ADA, NT5C, and XOR suggested increased purine catabolism to PNs and their derivatives upon fructose treatment.

To confirm the results of LC-MS and RNA-sequencing, PRPSAP1 and PPAT (rate-limiting enzymes from the first reaction in the pathway purine *de novo* synthesis) were chosen to identify gene and protein expression. Fructose administration increased the gene and protein expression of PRPSAP1 and PPAT in the livers of mice, and the increased fluorescence intensity of PRPSAP1 and PPAT confirmed this finding. Inconsistent mRNA and protein expression of PRPSAP1 and PPAT were observed, but this may have been due to differences between post-translational regulation and modification. With regard to how fructose accelerates purine *de novo* synthesis, mTOR has been shown to promote expression of the genes associated with PRPP synthesis and to regulate co-localization between purinosomes and mitochondria (14). We showed that fructose increased protein expression of P-mTOR, PRPSAP1, and PPAT significantly. In the presence of rapamycin (mTOR inhibitor), fructose did not affect the protein expression of PRPSAP1 or PPAT or the UA level in cell supernatants. The previous study also suggested that inhibition of mTOR expression could reduce fractional co-localization between purinosomes and mitochondria, thereby leading to a marked decrease in the metabolic flux through the purine *de novo* synthesis (14). Thus, fructose appears to accelerate the purine *de novo* synthesis by increasing mTOR expression. Besides, UA is a risk factor that can activate mTOR by inducing phosphorylation of protein kinase B (AKT) and proline-rich AKT substrate-40 (27). We suggest that the increased UA level caused by fructose intake may have a positive feedback effect on mTOR activation and purine *de novo* synthesis.

Furthermore, the effect of fructose intake on synthesis of AMP or GMP from IMP is not known. We found that fructose increased protein expression of Adss1 but decreased expression of IMPDH and GMPS significantly. These findings suggested that fructose intake mainly drives IMP to synthesize AMP, not GMP, which could be attributed to rapid depletion of ATP by fructose consumption (28). Fructose intake increased purine *de novo* synthesis as well as the PNs and their derivatives (adenine, xanthine, hypoxanthine). The increased gene expression and activity of XOR in the liver expedited the PNs degradation to UA. Normally, increased UA level can stimulate the expression of uricase, but reduced gene and protein expression of uricase caused by fructose intake was observed (Figure 6) in the present study. This phenomenon may be explained by uricase blockage increasing the metabolic rate of fructose and enhancing the ability of fructose to generate fat (29). Taken together, these results implied that fructose could inhibit the oxidation of UA to allantoic acid and increase the serum UA level.

## Conclusion

We demonstrated that fructose promoted purine *de novo* synthesis to generate IMP and drive conversion of IMP to AMP to maintain the rapid depletion of ATP. Fructose increased the breakdown of preformed PNs and their derivatives (adenine, xanthine, and hypoxanthine), accelerated their degradation to UA, and increased the serum UA level. This work revealed that increased purine *de novo* synthesis may be a crucial mechanism in fructose-induced hyperuricemia.

## Data availability statement

The data presented in the study are deposited here: https://doi.org/10.6084/m9.figshare.21701060.v1.

## Ethics statement

This animal study was reviewed and approved by Animal Care and Use Committee of Guangdong Medical University (Zhanjiang, China).

## Author contributions

YZ participated in the literature search, study design, surgery operation, data collection, data analysis, data interpretation, and manuscript writing. CZ carried out the data analysis and provided a critical revision of the manuscript. HS, WM, XC, YL, YZ, HZ, and YD conceived the study and participated in its design and coordination. All authors read and approved the final manuscript as the submitted version.

## Footnotes

1
http://geneontology.org/

2
http://www.genome.jp/

3
https://david.ncifcrf.gov/

4
http://www.biodeep.cn/

5
http://www.mzcloud.org/

6
https://cytoscape.org/

7
http://www.metaboanalyst.ca/
